# Acquiring Advanced Laparoscopic Colectomy Skills – The Issues

**DOI:** 10.21315/mjms2020.27.5.3

**Published:** 2020-10-27

**Authors:** Hizami Amin-Tai, Abdel Latif Khalifa Elnaim, Michael Pak Kai Wong, Ismail Sagap

**Affiliations:** 1 Department of Surgery, Universiti Putra Malaysia, Kuala Lumpur, Malaysia; 2 Kassala Police Hospital, Sudan; 3 School of Medical Sciences, Universiti Sains Malaysia, Kubang Kerian, Kelantan, Malaysia; 4 Universiti Kebangsaan Malaysia Medical Centre, Kuala Lumpur, Malaysia

**Keywords:** colorectal surgery, laparoscopic colectomy, learning curve, minimally invasive surgery, surgical education

## Abstract

Colorectal surgery has been revolutionised towards minimally invasive surgery with the emergence of enhanced recovery protocol after surgery initiatives. However, laparoscopic colectomy has yet to be widely adopted, due mainly to the steep learning curve. We aim to review and discuss the methods of overcoming these learning curves by accelerating the competency level of the trainees without compromising patient safety. To provide this mini review, we assessed 70 articles in PubMed that were found through a search comprised the keywords laparoscopic colectomy, minimal invasive colectomy, learning curve and surgical education. We found England’s Laparoscopic Colorectal National Training Programme (LAPCO-NTP) England to be by far the most structured programme established for colorectal surgeons, which involves pre-clinical and clinical phases that end with an assessment. For budding colorectal trainees, learning may be accelerated by simulator-based training to achieve laparoscopic dexterity coupled with an in-theatre proctorship by field experts. Task-specific checklists and video recordings are essential adjuncts to gauge progress and performance. As competency is established, careful case selections with the proctor are essential to maintain motivation and ensure safe performances. A structured programme to establish competency is vital to help both the proctor and trainee gauge real-time progress and performance. However, training systems both inside and outside the operating theatre (OT) are equally useful to achieve the desired performance.

## Introduction

Global population growth and ageing, along with the rising frequency of colorectal cancer (CRC) in the younger population, has added to the already existent burden on the healthcare system (1–5). In recent years, colorectal surgery has become more dynamic, thereby attracting the interest of many surgeons towards the field. This has been propagated by the rising incidence of CRC, which was reported as the third most commonly diagnosed cancer worldwide and the fourth leading cause of cancer deaths globally, with about 700,000 deaths per year, or 8% of cancer deaths, being attributed to CRC (3–5). CRC incidence has always been highest in developed nations and is observingly rising in developing countries (4, 6, 7).

Laparoscopic colectomy for CRC emerged in the early 1990s (8). Despite early reservations about applying laparoscopic techniques in colonic oncological resection, adequate evidence has been produced to show that laparoscopic colectomy produces similar oncological outcomes to open colectomy (9–12). Laparoscopic colectomy also has significantly less intraoperative blood loss, lower post-operative pain intensity, shorter post-operative ileus, shorter hospital stay and improved short-term quality of life (9–14). In addition, the laparoscopic technique may be more useful when compared to open resection in cases involving the extra-peritoneal rectum and intra-pelvic dissections (13, 15–17).

Unfortunately, the uptake of minimally invasive surgery (MIS) in colorectal operations has only gradually been advancing. For instance, there has generally been a slow progression of the technique. In the United States, MIS was employed in about 6.5% of colectomies in the early 2000s, and this number has increased to about 45% according to a survey conducted recently (18, 19). On the other hand, only 30% of colectomies are performed laparoscopically in England and Australia, while countries like South Korea, Japan and Singapore have reported that 60% of CRC cases employ MIS (20, 21). The adoption rates elsewhere in the world are much lower, with penetration rates lingering around 10% and most laparoscopic colectomies being performed in high-volume facilities and teaching centres (18–20, 22–24).

Unlike laparoscopic cholecystectomy or laparoscopic fundoplication, in which MIS techniques have been the gold standard of treatment, the adoption of laparoscopic colectomy by surgeons appears to have been hampered by factors such as higher equipment costs, the increased demand for operating room resources and longer operating times (20, 22, 23, 25, 26). The most common reasons for the reluctance to adopt laparoscopic colectomies among surgeons have been reported to be the long learning curve and the lack of formal training (20–22).

## Learning Curve in Laparoscopic Colectomy

**Figure 1:**
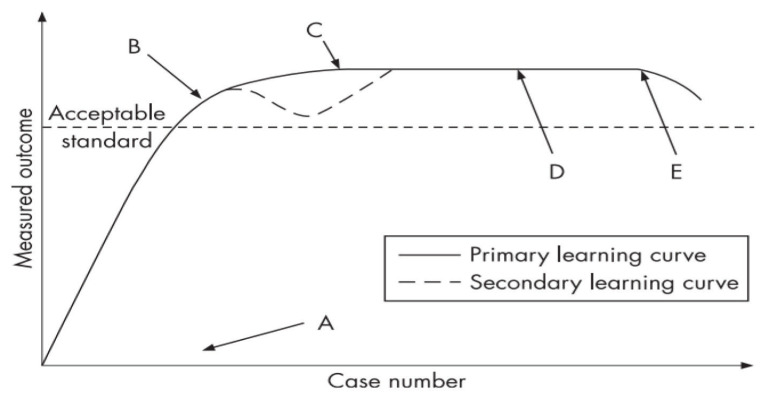
The idealised surgical learning curve (27) Notes: A = Commencement of training B = A point where the procedure can be performed independently and competently C = Small improvements in outcomes from further learning D = Plateau or asymptote E = Decline in performance due to advancing age (reduced dexterity, eyesight, memory, and cognition)

A learning curve is a plotted graph of performance against experience (Figure 1). In assessing a surgeon’s performance, measures of learning can be divided into two categories: i) measures of surgical process, or ii) a patient’s outcome (27). Surgical process measures include factors such as operative time, intra-operative complication rate, rate of conversion to open surgery in laparoscopic procedures and adequacy of oncological resection. Examples of patient outcome measures are the length of hospital stay, analgesia requirements, survival rate, morbidity rate and mortality rate. In cancer-related surgery, the most appropriate means of measuring learning would be improvements in case-adjusted long-term survival. However, such determinations require a long period of data collection before an analysis can be conducted. Moreover, incompetent surgeons are not likely be identified before it is too late, and much damage has already been done.

The reported number of cases needed to achieve competency ranges from 11 to 117 (Table 1). Most of these learning curve studies have used operative time and the rate of conversion as the yardsticks to assess progress in surgical skill performance. There have, however, been doubts that these two measures alone are suitable as learning curve parameters; this is because the majority of studies that produced lower numbers to achieve a plateau of the learning curves had a small sample size and adopted an inferior approach to statistical analysis (28). For example, in their report using the risk-adjusted cumulative sum (RA-CUSUM), Miskovic et al. (29) reported that a relatively high number of 150 performances was needed to achieve the plateau level of competence and this number was also reflected in other studies that employed similar statistical methods (29–31). It should also be noted that this high number was derived from individual studies where the surgeons were self-taught.

Colorectal surgery deals with heterogeneous clinical case presentations. As can be expected with protocols requiring advanced technological skills, adverse clinical outcomes during the early part of the learning process remain an ethical issue. Addressing this issue will not only alleviate concerns for patients; it will also help trainees to advance more quickly along the learning curve. An autodidactic approach to gaining competence in laparoscopic colectomy results is a protracted learning curve. By selecting the appropriate patient or case for the perceived skill level along with supervision by experienced trainers, a trainee should be able to safely hasten progress along the learning curve (29, 32–38).

## Out of OT Training

The prolonged learning curve for laparoscopic surgeries is due to the need to acquire specific sets of different skills to overcome the unique features and challenges (39). In laparoscopic colectomy, these challenges are further amplified due to the need to operate within multiple quadrants of the abdominal cavity, mobilisation of the bowel within a confined space, and dissection of inflamed or obliterated tissue planes (40). At the present time, trainees have become used to the fact that skills acumen should be gained faster. Self-learning is no longer acceptable and the apprentice-based learning model is too time consuming and costly (41). A surgeon’s experience is also an independent factor contributing to operative complications. Hence, allowing independent performances at the early part of their learning curve raises several ethical issues, and this problem could probably be addressed with the use of simulators (37, 38, 42–44). Simulator training involves the use of a high-fidelity virtual reality simulator (VRS), a box trainer, and animal, cadaveric, or synthetic material produced by three-dimension printing. These simulators are not solely employed to assist in achieving competence in surgical techniques, they also promote appreciation of anatomy and the plane of dissection. Simulation-based training (SBT) emulates a safe, controlled environment for trainees to practice within. Some methods even allow for reproducible conditions, enabling surgeons to train on a specific skill repeatedly. SBT is also less constrained by time or case availability compared to training within a real-life operating theatre (OT) setting.

**Figure 2:**
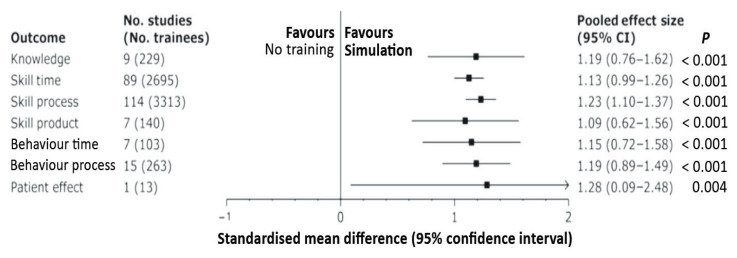
Simulation versus no intervention for laparoscopic training (45) Notes: Collated results of various studies comparing different types of outcomes such as knowledge outcomes, time outcomes, process outcomes, product outcomes, and patient outcomes between subject who had simulation-based training of laparoscopic skills and those who did not have any additional training. Reported results were converted to a standardised mean difference (Hedge’s g effect size). All effect size significantly favoured simulation-based training, regardless of outcome, level of learner, study design and the type of laparoscopic task trained

Studies comparing SBT against the absence of any additional training have shown that there has been significant improvement in all aspects of the outcomes tested (Figure 2) (45, 46). SBT has also been shown to be more effective than learning using video-based instructions. The use of a VRS has been shown to improve the real-life performance of surgical trainees while also being usable as a box trainer (45, 47–49). The use of a box trainer also seemed to help trainees acquire specific skills at a faster pace when compared to VRS. However, the addition of haptics or force feedback sensation did not improve training outcomes.

In addition to the abovementioned assistance, cadaveric or live-animal workshops have been extensively used for surgical training. Live-animal workshop tend to afford better quality and colour of organs, but the cadaveric workshop is by far a more superior training model for anatomical knowledge and realistic port placement (50). However, it was found that a similar tactile sensation was obtained in both types of training workshops and most participants felt that the experience gained was far more beneficial than observing cases in the OT (50).

Understanding various human anatomical planes and spaces is important in the performance of any surgery. However, this becomes more challenging when surgeons attempt to adopt laparoscopic colorectal techniques; therefore, the use of animal models to train for laparoscopic colorectal procedures may not be suitable. A similar conclusion can be derived from studies comparing box trainers and high-fidelity VRSs with cadaveric workshops. Both box trainers and VRSs are acceptable for attaining basic laparoscopic skills, but in a more complex procedure, such as laparoscopic colectomy, more improvements are required in terms of tissue realism and haptic feedback. Cadavers are better training models for all competency grades of trainees with respect to all complexity levels of procedures and they have been rated higher in terms of tissue reality and haptic feedback compared to VRSs or box trainers (45, 50–52).

## In OT Training

Attending a laparoscopic course alone is insufficient to gain proficiency in laparoscopic procedures and surgeons who do not commit to further training after attending a laparoscopic course are three times more likely to encounter complications (53). It has been noted that the presence of experienced supervisors is crucial during the early part of a trainee’s learning curve for laparoscopic colectomy. Apart from a longer operating time, the risks that patients are exposed to during a supervised session are quite similar to the risks that they encounter when being operated on by an expert. Furthermore, the oncological outcomes and mortality rates were also found to be comparable (32, 33, 54–56). Accordingly, surgical fellowship programmes are the current gold standard in the apprenticeship model of surgical training. These programmes have generally had a positive impact on patients’ outcomes. Surgeons who underwent fellowship training have produced better oncological resection and their patients tend to get discharged earlier than patients treated by surgeons who did not go through such a programme. Centres affiliated with fellowship programmes also produce lower complication and mortality rates (57). Nevertheless, the availability of fellowship programmes is limited, especially in developing nations such as Malaysia. An outreach training model is more realistic if the uptake of laparoscopic colectomy to be increased. An outreach programme minimises the negative effect on training opportunities for surgical trainees at fellowship training centres while providing training that is focused on the needs and available equipment of the centres involved in the outreach programme (22, 58, 59). Data comparing surgical complications and patients’ outcomes between colorectal fellows in training centres and surgeons engaged in outreach programmes have yielded similar results (59). These realisations have been the foundation of England’s Laparoscopic Colorectal National Training Programme (LAPCO-NTP) and this particular course of study has resulted in an increased uptake of laparoscopic colectomy from 5% in 2005 to 23% in 2009 (21, 58, 59).

## Case Selection for Learning Laparoscopic Colectomy

Conversion to open surgery is not a failure. It is regarded as a necessity when the technical limitations of laparoscopic surgery are reached, and such an approach may actually be indicative of a higher level of insight and the safe clinical judgement of a surgeon. However, conversion does bring about certain morbidities, such as longer operative time, increased wound complications and extended hospital stay (60). Realising a patient’s risk of conversion may guide a training surgeon on choosing appropriate cases to operate on or to convert earlier, especially when performing surgeries independently after completing the initial supervised training period. This would reduce unnecessary complications. Table 2 lists some of the independent factors that influence the possibility of conversion, such as the patient’s American Society of Anesthesiologists (ASA) physical status classification system, the patient’s body mass index (BMI), the type of surgery performed (Table 3), the presence of intra-abdominal abscess or fistula and the surgeon’s level of experience (38, 42–44, 53). An example of a validated prediction model for laparoscopic conversion risk is the Cleveland Clinic Foundation (CCF) Colorectal Laparoscopic Conversion (CLC) model (Table 4) (43). Cases can be stratified into increasing complexity levels based on their conversion risk and the model can act as a framework for case selection on the part of surgeons as they progress along their learning curve (29).

## Surgical Performance Assessment

Assessment of progress and testing whether the expert phase of the learning curve has been achieved are key elements in facilitating learning (27). A proficiently performed surgery involves 75% decision making and 25% operative performance, the latter of which includes some measure of dexterity (61). However, in laparoscopic colectomy, dexterity loss influences the entire operative process. Traditionally, surgical trainee assessment has relied on knowledge-based examinations, a logbook summarising the number of procedures performed, subjective senior evaluation and completion of compulsory training courses. These methods lack objectivity and may be vulnerable to bias. Operative dexterity is also rarely assessed. Operative skill may also be evaluated using unsuitable criteria, such as operative duration or the rate of conversion (62). An assessment system for surgical precision that is objective, unbiased, specific and sensitive should be employed. A task-specific checklist coupled with a global rating scale can ensure objectivity during the in-theatre assessment. The checklist also acts as a framework for trainees to use as part of their self-assessment. However, operating conditions vary with each surgery. Thus, laboratory or simulator-based training provides a more homogenous environment for repeated assessments. Various methods to this effect have been employed and validated, such as the Objective Structured Assessment of Technical Skills (OSATS), or dexterity analysis systems like the Imperial College Surgical Assessment Device (ISCAD) and VRS-based assessment (63, 64). A reliable assessment system can complement knowledge-based examinations, assist with training and provide a benchmark for certification (62).

## Conclusion

The adoption rate of laparoscopic colectomy is still low in developing nations due to the difficult and long learning curve. The exact number of surgeries required to obtain competency is still vaguely determined, but it could be estimated to be between 50 and 70 cases. An outreach programme such as that of England’s LAPCO-NTP could be used to motivate developing nations like Malaysia. Such a programme is probably the best option for surgeons to gain experience without compromising on patients’ safety. However, due to the low numbers of qualified colorectal surgeons, the target participants should be younger general surgeons who are competent in basic laparoscopic operations to disseminate such technical expertise on a wider scale. In Malaysia, the current national subspecialty training programme for colorectal surgery is proctorship-oriented, whereby the fellows receive extensive training in open and laparoscopic colectomy over 36 months. A balance of a structured training programme with proctorship-oriented would be ideal.

The fundamental aspects of learning can be accelerated by using simulator-based training to promote laparoscopic dexterity and in-theatre supervision by field experts. Strong proctorships from local experts acting as supervisors are best to shorten the skills acumen period. Task-specific checklists and video recordings are adjuncts that can be used by trainees to aid progress. Once independent practice is undertaken, careful case selection will help trainee surgeons’ progress along the learning curve in a faster and safer way.

## Figures and Tables

**Table 1:** Learning curve in laparoscopic colectormy

References	Number of patients	Outcomes measured	Average case per surgeon	Learning curve
Schlachta et al. (26)	461	OT IOCR CTO LOS POCR	154	30
† Tekkis et al. (31)	900	CTO OT POCR RA30	225	RS = 55 LS = 62 (total 117)
Kayano et al. (65)	250	CTO OT POCR	250	50
Simons et al. (66)	144	OT	36	11–15
† Dinçler et al. (30)	715	OT CTO IOCR POCR	362	70
Agachan et al. (67)	175	OT CTO POCR LOS	44	70
Bennett et al. (68)	1194	IOCR POCR	10	40
Tsai et al. (69)	240	OT BL LOS	240	RS = 15 SC = 15 LAR = 22
† Choi et al. (70)	199	IOCR CTO LN	66	SC = 36
‡ Miskovic et al. (29)	4907	OT IOCR CTO LOS POCR	189	152

Notes:

† CUSUM analysis used;

‡ Multicenter meta-analysis using CUSUM methods;

OT = operating time; IOCR = intra-operative complication rate; POCR = post-operative complication rate; RA30 = readmission within 30 days; LOS = length of stay. LN = lymph node yield; BL = intra-operative blood loss; CTO = rate of conversion to open; RS = right sided; LS = left sided; LAR = low anterior resection; SC = sigmoid colectomy

**Table 2:** Recommendations for case selection during the learning curve (29)

	Complexity level			
	I	II	II	IV
BMI (kg/m^2^)	< 27.5	< 30	< 30	> 30
Resection	Colon	Female pelvic	Male pelvic	Any
Diagnosis				
> Cancer‡	< T3§	T3§	T3	T4
> Inflammatory	None	Uncomplicated¶	Complicated⌘	Emergency

Notes:

† Approximate case experience: I = 1–50; II = 51–100; III = 100–150; IV = > 150;

‡ Pre-operative staging;

§ Excluding transverse colon, proctocolectomy;

¶ No intra-abdominal abscess or fistula;

⌘ Intra-abdominal abscess or fistula, restorative resection for ulcerative colitis

**Table 3:** Different diagnosis reflecting the level of surgical complexity (29)

Complexity	Diagnosis	Median
Level I	Solitary benign polyp	0
	T1–2 cancer	10
Level II	Partial resection/stricturoplasty for Crohn’s stricture	30
	Elective uncomplicated diverticular (no abscess)	40
	Cancer after stenting	50
	Perforation/bleeding due to penetrating trauma	50
	T3 cancer	50
	Partial resection of Crohn’s fistula	55
	Tumour in transverse colon	55
Level III	Acute bleeding	64
	Polyposis (e.g. FAP)	70
	Elective diverticular (with abscess on CT)	70
	Non-obstructive acute inflammation with perforation	73
	Restorative resection of ulcerative colitis	75
	Elective diverticular with colovesical fistula	75
Level IV	T4 cancer	80
	Acute obstruction	85
	Cancer with complicated fistula (e.g. colovesical)	90

Notes: Scale ranged from 0 (least complexity) to 100 (highest complexity). Complexity was split to four levels according to quartiles calculated from original data (30, 60, 80)

**Table 4:** CCF CLC model (43)

Risk factor	CCF CLC score	Total CLC score	Predicted conversion rate (%)
**ASA Class**			
1–2	0	0	0.2
3	1.2	0.5	0.4
4–5	1.8	1.0	0.6
**BMI**			
< 22	0	2.0	1.8
22–25	0.3	2.5	2.9
25.1–28.5	0.5	3.0	4.7
> 28.5	0.8	3.5	7.6
**Type of surgery**		4.0	11.9
Small bowel procedure/other	0	4.5	18.2
Abdominal rectopexy	0.9	5.0	26.9
Right-sided colonic resection	1.0	5.5	37.8
Left-sided colonc resection	1.6	6.0	50
Low rectal resection	2.2	6.5	62.2
Intra-abdominal Abscess		7.0	73.1
No	0	7.5	81.8
Yes	1.3	8.0	88.1
**Intra-abdominal fistula**			
No	0		
Yes	1.6		
**Surgeon seniority**			
Junior (*n* = 3)	0.4		
Senior (*n* = 2)	0		
